# Post-cardiopulmonary bypass hypoxaemia in paediatric patients undergoing congenital heart disease surgery: risk factors, features, and postoperative pulmonary complications

**DOI:** 10.1186/s12872-022-02838-9

**Published:** 2022-09-30

**Authors:** Yuan Sun, Xiao-Ming Deng, Ying Cai, Sai-E Shen, Li-Ya Dong

**Affiliations:** 1 Department of Anesthesiology and Critical Care Medicine, Xin Hua Hospital, Jiaotong University School of Medicine, No. 1665 Kongjiang Rd., Shanghai, 200092 China; 2 Department of Anesthesiology and Intensive Care Medicine, Changhai Hospital affiliated to Naval Medical University, Shanghai, 200438 China; 3 Department of Cardiothoracic Surgery, Xin Hua Hospital, Jiaotong University School of Medicine, No. 1665 Kongjiang Rd., Shanghai, 20092 China

**Keywords:** Congenital heart disease, Cardiopulmonary bypass, Hypoxaemia, Complications, Child

## Abstract

**Background:**

Hypoxemia after cardiopulmonary bypass (CPB) is the quantifiable manifestation of pulmonary dysfunction. This retrospective study was designed to investigate the risk factors for post-cardiopulmonary bypass hypoxaemia and the features of hypoxaemia and pulmonary complications in paediatric congenital heart disease surgery involving CPB.

**Methods:**

Data including demographics, preoperative pulmonary or cardiac parameters, and intraoperative interventions were retrospectively collected from 318 paediatric patients who underwent radical surgery with CPB for congenital heart disease. Among them, the factors that were significant by univariate analysis were screened for multivariate Cox regression. The lowest ratio of arterial oxygen tension and the inspiratory oxygen fraction (PaO_2_/FiO_2_), hypoxaemia (PaO_2_/FiO_2_ ≤ 300) insult time, duration of hypoxaemia, extubation time, and pulmonary complications were also analysed postoperatively.

**Results:**

The morbidity of post-cardiopulmonary bypass hypoxaemia was 48.4% (154/318). Months (6 < months ≤ 12, 12 < months ≤ 36 and 36 < months compared with 0 ≤ months ≤ 6: HR 0.582, 95% CI 0.388–0.873; HR 0.398, 95% CI 0.251–0.632; HR 0.336, 95% CI 0.197–0.574, respectively; *p* < 0.01), preoperative intracardiac right-to-left shunting (HR 1.729, 95% CI 1.200–2.493, *p* = 0.003) and intraoperative pleural cavity entry (HR 1.582, 95% CI 1.128–2.219, *p* = 0.008) were identified as independent risk factors for the development of post-cardiopulmonary bypass hypoxaemia. Most hypoxaemia cases (83.8%, 129/154) occurred within 2 h, and the rate of moderate hypoxaemia (100 < PaO_2_/FiO_2_ ≤ 200) was 60.4% (93/154).

**Conclusion:**

The morbidity of post-cardiopulmonary bypass hypoxaemia in paediatric congenital heart disease surgery was considerably high. Most hypoxaemia cases were moderate and occurred in the early period after CPB. Scrupulous management should be employed for younger infants or children with preoperative intracardiac right-to-left shunting or intraoperative pleural cavity entry.

## Introduction

Postoperative pulmonary dysfunction is a well-known cause of mobility and mortality after cardiac surgery with cardiopulmonary bypass (CPB) [1–3]. Although technologies involving CPB have improved, the incidence of various lung dysfunctions in patients undergoing cardiac surgery with CPB is still approximately 20–25% [4, 5].

Hypoxemia after CPB may result from primary pulmonary dysfunction, secondary cardiac dysfunction, or both. Hypoxemia is the quantifiable manifestation of pulmonary dysfunction that can be detected easily. The degree of hypoxaemia, which is defined as the ratio of arterial oxygen tension to the inspiratory oxygen fraction (PaO_2_/FiO_2_) with different PEEP levels, combined with the clinical insult time, chest imaging, and origin of oedema are the quaternion of acute respiratory distress syndrome (ARDS) [6]. In the definition of paediatric ARDS (PARDS) [7], PaO_2_/FiO_2_ ≤ 300 is one of the initial cut-off points of the abnormality in oxygenation. Although there is no consensual definition of hypoxaemia, the European Society of Anaesthesiology (ESA) and the European Society of Intensive Care Medicine (ESICM) [8] have unanimously defined hypoxaemia as a PaO_2_/FiO_2_ ≤ 300 mmHg and have recommended the preoperative and periprocedural use of noninvasive respiratory support techniques for these patients.

In studies using adult patients [9, 10] undergoing cardiac surgery, numerous perioperative risk factors for hypoxaemia have been reported. Compared to adults undergoing CPB, paediatric patients with congenital heart disease (CHD) differ greatly in the disease category, basal haemodynamics, postoperative pathophysiology changes, techniques and materials of CPB. Pulmonary complications of CHD occur due to structural compression, which causes paediatric airway malacia or atelectasis of the lung preoperatively, structural trauma to the respiratory system, damage to the alveolar-capillary membrane, and pulmonary oedema contributed by CPB [11]. All of these factors may lead to poorly compliant lungs that can deteriorate and cause hypoxaemia. Therefore, we hypothesized that several factors in paediatric surgery with CPB were involved specially in development of post-cardiopulmonary bypass hypoxaemia in this retrospective study. The aim of this study was to investigate the hypothesis, and to explore the features of the hypoxaemia, pulmonary complications after the surgery.

## Methods

### Patients

Between January 2015 and March 2016, there were 390 consecutive paediatric patients (≤ 12 years) scheduled for cardiac surgery with congenital heart disease. Based on the electronic medical system, we retrospectively screened the clinical, laboratory, and imageological data. Finally, 318 patients who underwent radical surgery therapy through median sternotomy with CPB were recruited into this study. The exclusion criteria were as follows: (1) surgery without CPB (ligation of patent ductus arteriosus, repair of coarctation of the aorta), (2) palliative surgery (pulmonary artery banding, systemic-to-pulmonary shunting), (3) hypoplastic left heart syndrome (HLHS) and single ventricle, (4) preoperative respiratory dysfunction (supported by mechanical ventilator), (5) residual intracardiac shunting from the right to the left (R to L) diagnosed by transoesophageal ultrasonic examination (TEE) intraoperatively or by echocardiography examination postoperatively, (6) postoperative left heart insufficiency (EF < 50%) as confirmed by echocardiography, and (7) any relevant clinical, laboratory or iconography data missing.

This observational study was approved by the Ethics Committee of Xinhua Hospital Affiliated to Shanghai Jiaotong University School of Medicine (approval No. XHEC-D-2017–006). Data were observed and recorded by health care personnel, including the nurses and medical doctors taking care of the patients. The study protocol was performed in accordance with the relevant guidelines, and informed consent was waived.

### Treatment regimen

All intraoperative and postoperative processes followed the routine protocol of our institution. Anaesthesia of all patients was induced by intravenous administration of midazolam, fentanyl, propofol, and rocuronium and maintained using midazolam, fentanyl, propofol, rocuronium, and sevoflurane. Mechanical ventilation was performed by a Grager Primus ventilator (Grager, Lubeck, Germany). The ventilator parameters were oxygen concentration of 40–60% (air-oxygen mixed), volume-controlled ventilation (VCV) mode: tidal volume of 10 ml kg^−1^ or pressure-controlled ventilation (PCV) mode: pressure limit of 12–15 cmH_2_O, ventilation frequency of 16–28 to maintain the P_ET_CO_2_ level at 4.5–5.5 kPa, and I/E ratio of 1/2. In addition to routine monitors, invasive artery pressure monitoring and blood-gas samples were obtained via an arterial line in the radial or femoral artery.

CPB was carried out with a Maquel Jostra Cardiopulmonary HL20 (Maquel, Germany). Management of CPB included systemic temperature drift to 33–35 °C, pump flow rates of 150–200 ml kg^−1^ min^−1^, mean perfusion pressure between 30 and 60 mmHg, haematocrit between 20 and 30%, a crystal-colloid proportion of the priming solution that was 0.5:1, albumin and plasma priming to maintain the colloid osmotic pressure at 17–19 mmHg, and conventional combined modified ultrafiltration.

Mechanical ventilation was terminated during CPB. Before weaning from CPB and sternal closure, the lungs were re-expanded by the following conventional alveolar recruitment manoeuvre: peak airway pressure of 20–30 cmH_2_O for three to five consecutive mechanical breaths. Mediastinal drainage connected to continuous negative pressure suction was performed.

The surgical effect and cardiovascular function were routinely evaluated by an iE33 ultrasound device (Philips, Bothell WA, USA) connected to a TEE probe (S7-3t) before weaning from CPB and the transthoracic probe (S5-1) within 24 h postoperatively in the ICU.

The arterial blood-gas analyses were repeated after anaesthetic induction, after weaning from CPB, before transport out of the OR, every 2 h before extubation, and every 4 h after extubation until discharge from the ICU. All patients were routinely examined by thoracic X-ray after admission to the ICU within 8 h after the operation. Thoracic X-ray was repeated, and scanning by computed tomography (CT) was performed whenever necessary.

In the ICU, ventilator setting adjustments were guided by blood-gas analyses and imaging examinations. The timing of extubation and discharge from the ICU was decided according to the haemodynamic, respiratory, and neurological status and other related clinical parameters.

### Clinical data collection and examination method

According to the results of adult’s studies [9, 10] and the particularity of paediatric patients with CHD undergoing CPB [11], we hypothesized that several perioperative factors might affect respiratory function after CPB. They were retrospectively collected from the electronic medical record system. These factors included age (months), sex, weight, recent upper respiratory tract infection history (yes/no, Y/N; URI, defined as within seven days preoperatively), history of cardiac operation (Y/N), preoperative intracardiac R-to-L shunt (Y/N), preoperative pulmonary hypertension (Y/N), preoperative left ventricular ejection fraction, Aristotle basic complexity score (ABC) category [12], duration of CPB, duration of aortic cross-clamping, ventilation mode during the operation (PCV/VCV), the total blood products used intraoperatively and postoperatively transfused (including CPB priming), and pleural cavity entry during the operation (Y/N).

The PaO_2_/FiO_2_ value by arterial blood-gas analyses at each time point from termination of CPB to discharge from the ICU was used to measure hypoxaemia. The time points were after weaning from CPB and the completion of ultrafiltration, before transport out of the OR, every 2 h before extubation, and every 4 h after extubation in the ICU. The lowest PaO_2_/FiO_2_ ≤ 300 was defined as hypoxaemia [8]; 200 < PaO_2_/FiO_2_ ≤ 300 was defined as mild hypoxaemia, 100 < PaO_2_/FiO_2_ ≤ 200 was defined as moderate hypoxaemia, and PaO_2_/FiO_2_ ≤ 100 was defined as severe hypoxaemia [13]. The features of hypoxaemia, including the hypoxaemia degree, hypoxaemia insult time [defined as the earliest time point (hours after the weaning from CPB and completion of ultrafiltration) of hypoxaemia occurrence] and duration of hypoxaemia, were collected. The endpoint of follow-up to hypoxaemia was the discharge time from the ICU. Survival to hypoxaemia referred to the interval from the endpoint of CPB and ultrafiltration to the hypoxaemia insult time or the last follow-up.

Furthermore, the postoperative pulmonary outcomes of all patients were also screened as follows: extubation time (hours after operation), radiographic evidence and pulmonary complications. Pulmonary complications were diagnosed by clinical symptoms and/or radiographic evidence (X-ray or CT) according to the criteria for congenital heart disease: [11, 14] including chylothorax, pleural effusion, pneumonia, pneumothorax, atelectasis, postoperative respiratory insufficiency requiring mechanical ventilatory support > 7 days, postoperative respiratory insufficiency requiring reintubation, and postoperative respiratory failure requiring tracheostomy.

### Statistical analysis

All analyses were performed using IBM SPSS Statistics (version 21.0, IBM, Almonk, NK). Continuous variables were screened by the Kolmogorov–Smirnov one-sample test. The results are expressed as the median [interquartile range (IQR)] for nonnormally distributed data and as the mean ± SD for normally distributed data. The frequency variables are expressed as numbers (%). The Mann‒Whitney U test was used for the comparison of continuous variables, while Pearson’s chi-square or Fisher’s exact test was used for univariate analysis. Factors that were significantly different (*p* < 0.1) in the post-cardiopulmonary bypass hypoxaemia incidence shown by univariate analysis were used for multivariate Cox regression analysis (backwards) [hazard ratio (HR) and 95% confidence interval (CI)] to determine the independent factors associated with post-cardiopulmonary bypass hypoxaemia. Spearman analysis was used for the bivariate correlations. All statistical hypothesis tests were two-sided and performed at the 0.05 significance level. *p* < 0.05 was considered statistically significant.

## Results

### Study population

**Fig. 1:**
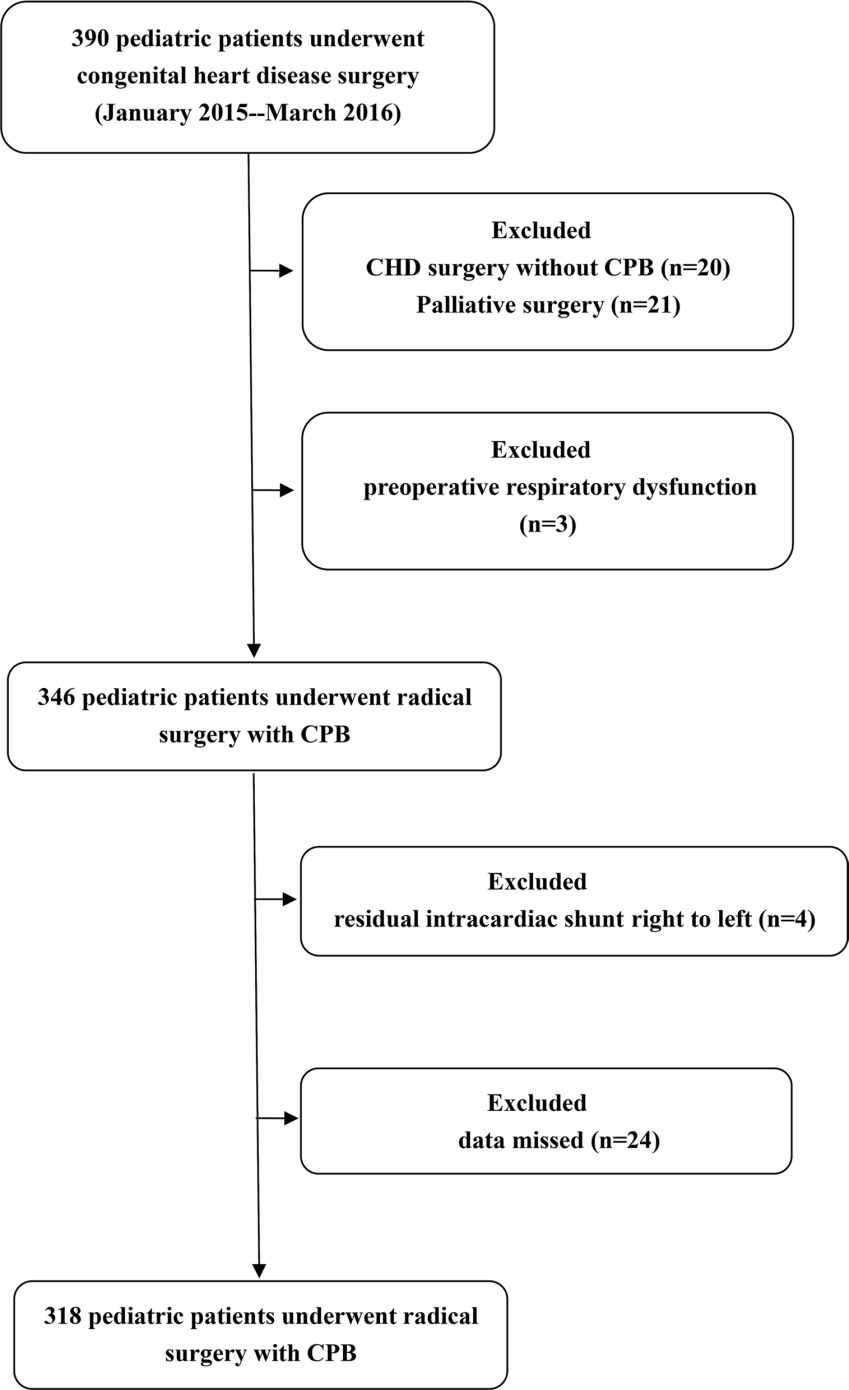
Flowchart of the patient’s enrollment

A total of 318 paediatric patients were retrospectively enrolled in this study. Figure 1 shows the flow chart of inclusion and exclusion. The demographic and perioperative data are reported in Table 1.

**Table 1:** The demographic and perioperative data of the patients

	N = 318
Disease category	
Atrial or ventricular septal defect (or with valvular disease)	240(75.5%)
Tetralogy of Fallot	53(16.7%)
Anomalous pulmonary venous drainage	7(2.2%)
Endocardial cushion defect	5(1.6%)
Aortic valve disease	3(0.9%)
Mitral valve disease	3(0.9%)
Double outlet of right ventricle	3(0.9%)
Pulmonary valve stenosis	2(0.6%)
Ebstein anomaly	1(0.3%)
Coronary sinus fistula	1(0.3%)
Age, months	12(6–36)
0 ≤ months ≤ 6	69(21.7%)
6 < months ≤ 12	76(23.9%)
12 < months ≤ 36	87(27.4%)
36 < months	86(27.0%)
Weight (kg)	9.5(7–14)
Gender male/female	175/143
History of cardiac operation	19(6.0%)
Recent URI	47(14.8%)
Pulmonary hypertension	131(41.2%)
Intracardiac shunt R to L	141(44.3%)
Left ventricular ejection fraction%	65(62–68)
Aristotle basic complexity score	
Category 1	29(9.1%)
Category 2	260(81.8%)
Category 3	27(8.5%)
Category 4	2(0.6%)
Duration of CPB, min	57(48.0–81.3)
Duration of aortic cross-clamping, min	30(23.0–46.3)
Transfusion volume, ml	500(500–600)
Ventilation mode PCV/VCV	96/222
Pleural cavity entry	171(53.6%)

Data are presented as media IQR), number %)

### Risk factors for post-cardiopulmonary bypass hypoxaemia

The overall incidence of post-cardiopulmonary bypass hypoxaemia was 48.4% (154/318). Potential risk factors for post-cardiopulmonary bypass hypoxaemia identified by univariate analysis are shown in Table 2. The difference in hypoxaemia incidence for the factors including age (months), weight, recent URI, pulmonary hypertension, preoperative intracardiac R-to-L shunt, transfusion volume, intraoperative ventilation mode (PCV), and pleural cavity entry were statistically significant (*p* < 0.1). Next, the abovementioned related univariate factors (except weight, because of the linear interactive effect between weight and month rank in terms of paediatric physical growth) were analysed for multivariate Cox regression statistics. The results are shown in Table 3.

**Table 2:** Univariate analysis of possible risk factors for the post-cardiopulmonary bypass hypoxemia

	Hypoxemia (+)	Hypoxemia (−)	*P* value
	(n = 154)	(n = 164)	
Age(months)			< 0.001*
0 ≤ months ≤ 6	61 (39.6%)	8 (4.9%)	
6 < months ≤ 12	42 (27.3%)	34 (20.7%)	
12 < months ≤ 36	30 (19.5%)	57 (34.8%)	
36 < months	21 (13.6%)	65 (39.6%)	
Weight (kg)	7 (5.95–10)	12 (9–17.5)	< 0.001*
Gender male/female	86/68	89/75	0.778
History of cardiac operation	6 (3.9%)	13 (7.9%)	0.13
Recent URI	32 (20.8%)	15 (9.1%)	0.003*
Pulmonary hypertension	88 (57.1%)	43 (26.2%)	< 0.001*
Intracardiac shunt R to L	99 (64.3%)	42 (25.6%)	< 0.001*
Left ventricular ejection fraction%	64.00 (60.00–68.00)	65.00 (62.00–68.00)	0.310
Aristotle basic complexity score			0.631
Category 1	13 (8.4%)	16 (9.8%)	
Category 2	129 (83.8%)	131 (79.9%)	
Category 3	12 (7.8%)	15 (9.1%)	
Category 4	0 (0%)	2 (1.2%)	
Duration of CPB, min	61.00 (51.00–82.00)	54.00 (45.00–79.25)	0.003^*^
Duration of aortic cross-clamping, min	33.00 (27.00–48.25)	27.00 (21.00–44.75)	0.003^*^
Transfusion volume, ml	500.00 (500.00–600.00)	500.00 (500.00–600.00)	0.011^*^
Ventilation mode PCV	59 (38.3%)	37 (22.6%)	0.002^*^
Pleural cavity entry	101 (65.6%)	70 (42.7%)	< 0.001^*^

Recent URI: upper respiratory tract infection within seven days preoperatively

Data are presented as media (IQR), number (%), number. *p < 0.05 (significant)

**Table 3:** Multivariate Cox regression for analyzing of risk factors for the post-cardioplumonary bypass hypoxemia

	Regression coefficient	SE	HR	95% CI	*P* value
Months					< 0.001^*^
0 ≤ months ≤ 6			1 (reference)		
6 < months ≤ 12	− 0.542	0.207	0.582	0.388–0.873	0.009^*^
12 < months ≤ 36	− 0.922	0.236	0.398	0.251–0.632	< 0.001^*^
36 < months	− 1.090	0.272	0.336	0.197–0.574	< 0.001^*^
Recent URI	0.031	0.212	1.032	0.680–1.565	0.883
Pulmonary hypertension	0.328	0.182	1.388	0.972–1.981	0.071
Intracardiac shunt R to L	0.548	0.187	1.729	1.200–2.493	0.003^*^
Ventilate mode PCV	0.075	0.171	1.078	0.771–1.507	0.661
Duration of CPB	0.000	0.003	1.000	0.994–1.006	0.957
Duration of aortic cross-clamping	0.000	0.011	1.000	0.978–1.023	0.989
Transfusion volume	0.000	0.000	1.000	0.999–1.001	0.821
Pleural cavity rupture	0.459	0.173	1.582	1.128–2.219	0.008^*^

*p < 0.05 (significant)

The months (6 < months ≤ 12, 12 < months ≤ 36 and 36 < months compared with 0 ≤ months ≤ 6: HR 0.582, 95% CI 0.388–0.873; HR 0.398, 95% CI 0.251–0.632; HR 0.336, 95% CI 0.197–0.574, respectively; *p* < 0.01), preoperative intracardiac R-to-L shunting (HR 1.729, 95% CI 1.200–2.493, *p* = 0.003) and intraoperative pleural cavity entry (HR 1.582, 95% CI 1.128–2.219, *p* = 0.008) were identified as independent risk factors for the development of post-cardiopulmonary bypass hypoxaemia.

### Features of hypoxaemia

**Fig. 2:**
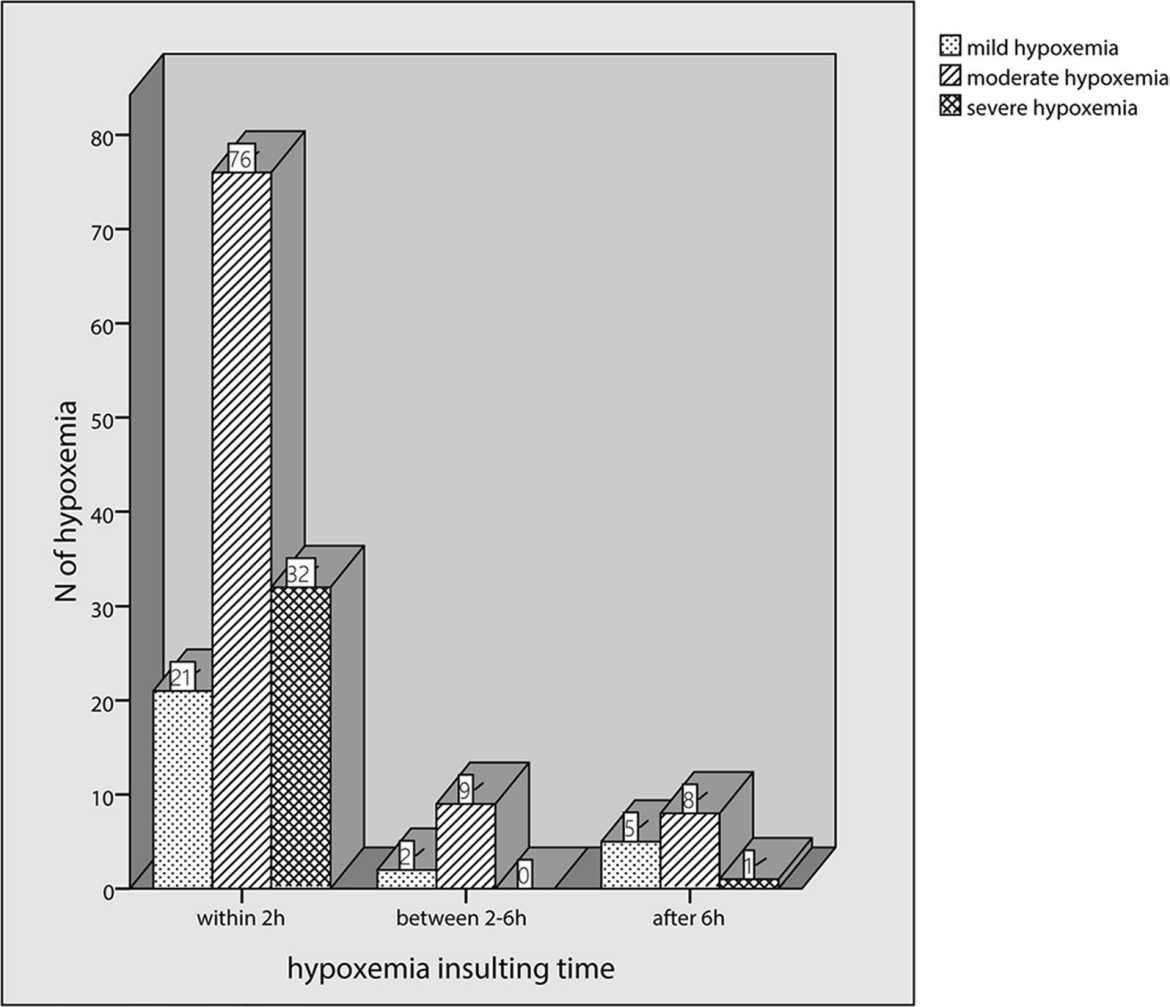
The number and degree of the hypoxemia cases in different periods

Among all 154 paediatric patients with post-cardiopulmonary bypass hypoxaemia, the hypoxaemia insult time (after CPB) was 0 (0–0) hours. The hypoxaemia duration was 38.0 (2.0–82.5) hours, and the lowest P/F value was 148.7 ± 54.3. There were 28 (18.2%) mild hypoxaemia cases, 93 (60.4%) moderate hypoxaemia cases, and 33 (21.4%) severe hypoxaemia cases. Most hypoxaemia cases (83.8%, 129/154) occurred within 2 h, while 11 (7.1%) cases occurred between 2–6 h, and 14 (9.1%) cases occurred after 6 h. The number and degree of hypoxaemia cases in different periods are shown in Fig. 2.

### Postoperative pulmonary outcomes

The extubation times (hours after operation) of hypoxaemia patients and nonhypoxaemia patients were 48.0 (20.5–104.0) and 6 (4.0–16.8), respectively. In hypoxaemia cases, the duration of hypoxaemia (*p* < 0.001, r = 0.831) and the degree of hypoxaemia (*p* = 0.001, r = 0.255) were positively correlated with the extubation time. Pulmonary complications postoperatively screened in the population of our study included pneumonia, atelectasis, pleural effusion, pneumothorax, postoperative respiratory insufficiency requiring mechanical ventilatory support > 7 days, and postoperative respiratory insufficiency requiring reintubation. Atelectasis (13.0% vs. 3.0%, p = 0.001), pleural effusion (13.0% vs. 6.1%, *p* = 0.036) and postoperative respiratory insufficiency requiring mechanical ventilatory support > 7 days (9.7% vs. 0%, *p* < 0.001) occurred more often in children with hypoxaemia than in those without hypoxaemia (Table 4). Eighty-eight (57.1%) hypoxaemic children had negative radiographic results, and 34 (20.7%) nonhypoxaemic children had positive radiography for pulmonary complications.

**Table 4:** Hypoxemia and the pulmonary complications

	hypoxemia (+) n = 154	Hypoxemia (−) n = 164	*P* value
Pneumonia	33 (21.4%)	27 (16.5%)	0.258
Atelectasis	20 (13.0%)	5 (3.0%)	0.001*
Pleural effusion	20 (13.0%)	10 (6.1%)	0.036*
Pneumothorax	4 (2.6%)	2 (1.2%)	0.437
Postoperative respiratory insufficiency requiring mechanical ventilatory support > 7 days	15 (9.7%)	0 (0%)	< 0.001*
Postoperative respiratory insufficiency requiring reintubation	4 (2.6%)	0 (0%)	0.055

Data are presented as number (%). *p < 0.05 (significant)

## Discussion

The pathophysiology of postoperative pulmonary dysfunction in CPB surgery is multifactorial and complicated [3], involving the specificity of cardiac surgery, alterations of lung mechanics in CPB, and anomalies in gas exchange. Reduced respiratory function may lead directly to a decreased PaO_2_/FiO_2_ ratio,or to pulmonary failure requiring prolonged mechanical ventilatory support and even to ARDS. In studies involving adult patients [10, 15, 16], the incidence of hypoxaemia (PaO_2_/FiO_2_ ≤ 200 as the marker) after CPB cardiac surgery has been reported to range from 30.6% to 54.2%. In a recent study [17], the morbidity of PaO_2_/FiO_2_ < 300 measured 2 h after CPB cessation was reported to be as high as 39.1%. Herein, we used PaO_2_/FiO_2_ ≤ 300 as the marker of hypoxaemia. Our analysis revealed that the overall incidence of post-cardiopulmonary bypass hypoxaemia in paediatric cardiac surgery was 48.4% (154/318).

Several preoperative, intraoperative, and postoperative factors have been identified as significant risk factors for hypoxaemia in adult CPB [9, 10]. In children, the respiratory centre is immature, and the stimuli of the CPB, surgical performance, and anaesthesia procedure decrease pulmonary surfactant. Abnormalities in gas exchange and increased respiratory resistance are common, including the increased alveolar-arterial oxygen partial pressure difference, elevated pulmonary vascular resistance, increased intrapulmonary shunt, and decreased compliance, especially in neonates and younger infants. Stayer et al. [18] reported that age was the only significant factor that affected both dynamic respiratory compliance and total respiratory resistance and was a stronger predictor of these changes in respiratory mechanics. The major cases in our study were infants (45.6%) and children of young age (12 < months ≤ 36: 27.4%). Our univariate analysis revealed that both age (months) and weight were significantly related to post-cardiopulmonary bypass hypoxaemia (*p* < 0.001). In the results of multivariate analysis, low age (months) was a risk factor for hypoxaemia, which was significantly decreased in older infants and children (6 < months ≤ 12, HR 0.582; 12 < months ≤ 36, HR 0.398; 36 < months, HR 0.336) compared with younger infants (0 ≤ months ≤ 6). Scrupulous perioperative management may be needed for younger children, especially younger infants (0 ≤ months ≤ 6), in order to alleviate deoxygenation.

Preexisting intracardiac R-to-L shunting was related to post-cardiopulmonary bypass hypoxaemia and was a significant predictor identified by univariate (*p* < 0.001) and multivariate (*p* < 0.001) analyses. Children with congenital heart disease with intracardiac right-to-left shunts were preoperatively found to have hypoxaemia to varying degrees. Although these children tolerated physiological hypoxaemia, they had numerous pathological changes, such as erythrocytosis, hypervolemia, pulmonary vasodilation, small-pulmonary-vessel and collateral-circulation hyperplasia, and alveolar hyperventilation combined with chronic respiratory alkalosis. Children who had chronic hypoxaemia over a long period had limited cardiac reserve and oxygen evolution capacity. These children might have had a higher blood dilution after CPB than the children who were neither anoxic nor had a right-to-left shunt. In addition, the proinflammatory cytokine interleukin [interleukin 6 (IL-6)] levels after CPB were inversely correlated with PaO_2_/FiO_2_ values 24 h postoperatively in infants. IL-6 was higher in the infants with hypoxaemia due to intracardiac right-to-left shunting in contrast to those with intracardiac left-to-right shunts preoperatively, during CPB and after CPB, whereas the natural anti-inflammatory cytokine IL-10 was lower preoperatively and during CPB [19]. Considering the disease categories in our study, there might be intracardiac R-to-L shunting in tetralogy of Fallot, anomalous pulmonary venous drainage, endocardial cushion defects, a double outlet of the right ventricle, pulmonary valve stenosis, Ebstein anomaly and even an atrial or ventricular septal defect with pulmonary vascular resistance close to the systemic vascular resistance. Children who are younger and are in more serious condition with the above diseases need urgent operative correction.

Median sternotomy and manipulation of the heart and great vessels may result in pleural cavity entry unilaterally or bilaterally. The disappearance of intrathoracic negative pressure may lead to alveolar collapse that radically reduces the aeration area. The fluid and blood filling the pleural cavity compress the pulmonary tissue and induce atelectasis. Although pleural cavity entry is almost always identified by surgeons in the later period of weaning from CPB, therapies including suction of the ruptured cavity, mediastinal drainage, and alveolar recruitment are performed conventionally. Small pneumothorax, atelectasis, or haemothorax may exist until the later postoperative period. Our study showed that intraoperative pleural cavity entry was a significant independent risk factor for hypoxaemia.

Our results showed that hypoxaemia in paediatric cardiac surgery occurred very early [0(0–0) hours] after CPB; in most cases (83.8%), it occurred within 2 h. This phenomenon was similar to that observed in adult patients [17], e.g., PaO_2_/FiO_2_ decreased significantly 2 h after CPB. Several factors may have contributed to the timing characteristics, as follows: the atelectasis produced in CPB explained most of the markedly increasing intrapulmonary shunting and hypoxaemia in the early post-CPB period (45 min after separation from CPB) [20]; the extravascular lung water increased by 52% after separation from CPB and decreased to the presurgical values 4 h postoperatively [21]; and the lung and chest wall mechanics were mainly affected from 20–30 min to 4 h after separation from CPB [22].

Hypoxemia is the most easily detected clinical manifestation of postoperative pulmonary dysfunction, as well as the result of pulmonary complications. Studies in adult patients showed that postoperative pulmonary dysfunctions after cardiac surgery included pleural effusion (27–95%), atelectasis (16.6–88%), pneumonia (0.9–4.4%), ARDS (0.5–1.7%), and postoperative hypoxaemia without any clinical symptoms (3–10%) [23]. In paediatric studies, there was a fairly high incidence of pulmonary complications after cardiac surgery, such as nosocomial pneumonia (9.6%-21.5%) [24, 25], chylothorax (3.8%) [26], and diaphragmatic paralysis due to phrenic nerve injury (5.4–17.6%) [27]. Our study explored the relationship between postoperative pulmonary complications and hypoxaemia. Among them, atelectasis, pleural effusion, and postoperative respiratory insufficiency requiring mechanical ventilatory support > 7 days were significantly related to hypoxaemia. More than half of the children with hypoxaemia (57.1%) were not diagnosed with abnormalities based on conventional radiographic evidence (bedside X-ray or CT). Meanwhile, 20.7% of nonhypoxaemic children had positive radiographic findings. In addition, bedside chest X-ray was reported to have a diagnostic accuracy of 47% for pleural effusion, 75% for alveolar consolidation, and 72% for the alveolar–interstitial syndrome, with chest CT being the diagnostic gold standard [28]. Even the ‘golden standard’ CT scanning was used and occasionally repeated as the routine examination, considering the expense and the difficulties in patients with mechanical ventilation.

Our results indicated that the majority of hypoxaemia cases were not positive by conventional radiographic examination; therefore, more effective diagnostic imaging tests for hypoxaemia and more vigorous therapy for improving oxygenation should be considered intraoperatively and in the early postoperative period. One such test is the highly sensitive, specific, and reproducible bedside pulmonary ultrasonogram [28], which can detect the early stage of extravascular water accumulation in the presence of a normal PaO_2_/FiO_2_ ratio [29]. Ventilatory support, including the alveolar recruitment manoeuvre, individualized optimal positive end-expiratory pressure (PEEP) as part of a lung-protective strategy, and other “nonconventional” mechanical ventilation [30, 31], may also be promising and reliable for these high-risk children.

The present study has the following limitations: (1) PaO_2_/FiO_2_ was used to qualitatively and quantitatively analyse post-cardiopulmonary bypass hypoxaemia. To exclude the interference of intracardiac shunting and abnormal pulmonary blood flow with the P/F value, we only enrolled patients whose anatomical abnormalities could be completely corrected. Thus, this population did not entirely cover children with surgical congenital heart disease. (2) The specific patients in our study had relatively small differences in surgical procedures, CPB techniques, and therapeutic algorithms employed in the ICU. There were not as many perioperative variables included for the risk analyses as in studies using adult patients. (3) This retrospective study was conducted at a single centre. The sample size was determined by the number of potential risk factors and was relatively small, thus limiting the power of the present study.

## Conclusion

The incidence of post-cardiopulmonary bypass hypoxaemia in paediatric congenital heart disease surgery is considerably high. Younger age, preoperative intracardiac R-to-L shunting, and intraoperative pleural cavity entry were identified as independent risk factors for hypoxaemia. Most hypoxaemia cases were moderate and occurred within 2 h. The postoperative pulmonary complications diagnosed by conventional radiographic evidence did not sensitively or specifically translate to hypoxaemia. Additional manoeuvres to optimize the paediatric patient's oxygenation may be performed perioperatively in high-risk populations to decrease the duration of mechanical ventilation and reduce pulmonary complications, morbidity and hospital costs.

## Data Availability

The datasets used and analyzed during the study are available from the corresponding authors on reasonable request.
